# Intramuscular immunization with a CpG-adjuvanted Cryptococcus neoformans vaccine induces sterilizing immune protection.

**DOI:** 10.21203/rs.3.rs-8603960/v1

**Published:** 2026-10-23

**Authors:** Yu Zhang, Yina Wang, Keyi Wang, Amariliz Rivera, Chaoyang Xue

## Abstract

Despite the rising burden of invasive fungal disease, no fungal vaccine is available to use. Cryptococcus neoformans is a fungal pathogen that initiates pulmonary infection and can disseminate to cause fatal meningoencephalitis. We previously identified HK-fbp1, a heat-inactivated C. neoformans mutant strain lacking the F-box protein Fbp1, that confers durable mucosal protection. Here, we combine HK-fbp1 with the type-C CpG adjuvant ODN2395, enabling effective intramuscular immunization to provide sterilizing immune protection against pulmonary challenge, with mostly complete clearance of fungal burden and blockade of extrapulmonary dissemination. Protection correlated with strong recruitment of innate immune cells to the lung and a coordinated CD4⁺ T-cell response. Cryptococcus-specific CD4⁺ T cells produced IFN-γ and IL-17A in lung-draining lymph nodes, whereas muscle-draining nodes exhibited strong IL-2, consistent with coordinated priming and Th1/Th17 effector programming. Protection required IFN-γ, but not other interferons, and persisted in CD4⁺ T-cell- or B-cell-deficient mice, supporting development for immunocompromised hosts. Together, these findings establish CpG-adjuvanted intramuscular HK-fbp1 vaccination as a clinically tractable strategy that can elicit sterilizing antifungal immunity and provide a foundation for advancing HK-fbp1-based antifungal vaccines.

